# No relative age effect in professional esports: a cross-national test of selection mechanisms

**DOI:** 10.3389/fpsyg.2026.1829930

**Published:** 2026-07-15

**Authors:** Jimoon Kang

**Affiliations:** School of Media and Communication, Korea University, Seoul, Republic of Korea

**Keywords:** birth quarter, career trajectory, cross-national quasi-experiment, debut age, esports, propensity score matching, relative age effect, survival analysis

## Abstract

**Background:**

The relative age effect (RAE) is well-documented in traditional sport, but its applicability to professional esports remains largely unexplored. The two existing studies examined only the entry question, without controlling for birth seasonality, testing causal mechanisms, or analyzing post-entry career trajectories.

**Objective:**

This study examined (1) whether RAE exists in professional esports after adjusting for birth seasonality; (2) whether academic cutoff dates causally influence esports career outcomes; (3) whether birth quarter affects career longevity and earnings after professional entry; and (4) whether birth quarter operates indirectly through debut age.

**Methods:**

The sample comprised 15,065 professional esports players with confirmed birthdates from the Esports Earnings database, spanning 177 countries and 609 game titles. Methods included chi-square tests with population-adjusted expected frequencies, a cross-national quasi-experiment exploiting cutoff variation across 20 countries, Kaplan–Meier survival analysis and Cox regression, propensity score matching, and Baron-Kenny mediation analysis with the Sobel test.

**Results:**

After adjusting for birth seasonality, no aggregate RAE was detected [χ^2^(3) = 1.38, *p* = 0.711]. A cohort reversal emerged: players born after 2006 exhibited a traditional RAE (OR = 1.92, *p* < 0.001), while those born in 1991–2000 showed an inverse RAE (OR = 0.81–0.85, *p* < 0.05). Cross-national analyses revealed no causal effect of cutoff dates on career outcomes. Post-entry, birth quarter had no direct effect on career duration (log-rank *p* = 0.593) or earnings (propensity-score-matched *p* = 0.487). Birth quarter operated indirectly through debut age: late-born players debuted 0.16 years earlier, mediating 48.5% of the total association with earnings (Sobel *z* = 2.57, *p* = 0.010).

**Conclusion:**

At the aggregate level, professional esports shows no relative age effect after adjusting for birth seasonality, and cross-national evidence is consistent with academic cutoff dates having no causal effect on career outcomes. Birth quarter is associated with career earnings only indirectly, through the timing of debut. The traditional RAE pattern emerging in the youngest cohort (born after 2006) may warrant attention as esports adopts more structured youth development systems.

## 1 Introduction

The common practice of grouping children and adolescents by chronological age for competitive sport has long been recognized as a double-edged sword. While age-based categorization is intended to create fair and developmentally appropriate competition, it introduces an unintended systematic bias: players born shortly after the selection cutoff date enjoy up to 11 months of additional physical, cognitive, and emotional development compared with peers born just before the cutoff. This developmental gap (trivial in adulthood but substantial during childhood and adolescence) confers measurable advantages in selection, participation, and performance. The resulting phenomenon, in which individuals born earlier in the selection year are overrepresented at higher levels of competition, is known as the relative age effect (RAE; Barnsley et al., 1985; Musch and Grondin, 2001).

Since its initial documentation in Canadian ice hockey (Barnsley et al., 1985), the RAE has been replicated across an extraordinary range of sports, countries, and competitive levels. Meta-analyses have confirmed pervasive overrepresentation of relatively older athletes (Cobley et al., 2009), with significant effects across female sport contexts (Smith et al., 2018) and persistent effects in soccer from youth to professional levels (Bozdìch et al., 2023). The phenomenon extends beyond physically demanding sports to cognitive-skill domains such as chess (Breznik and Law, 2016). Four decades after its initial discovery, recent editorial assessments characterize the RAE as one of the most robust findings in developmental sport science (Barnsley, 2024; Kelly et al., 2025).

Three interrelated mechanisms have been proposed to explain the RAE, though disentangling their individual contributions has proven methodologically challenging. The first and most frequently invoked mechanism is maturation-related selection bias. Relatively older children within an annual cohort tend to be taller, stronger, and more physically coordinated, traits that coaches and selectors preferentially identify as “talent” (Cobley et al., 2009; Musch and Grondin, 2001). Cross-national studies have demonstrated that this bias tracks administrative cutoff dates: when countries shift their selection cutoff, the birth-month advantage shifts accordingly (Musch and Hay, 1999; Helsen et al., 2005). Helsen et al. (2012) revisited the cross-European data a decade later and found the effect essentially unchanged, indicating that awareness alone had not resolved it.

The second mechanism is structural amplification through age-grouping systems. The cutoff date creates the initial maturation disparity; institutional structures then amplify it. Selected athletes receive better coaching, more competitive training environments, and greater playing time, advantages that compound across developmental years in a process akin to the Matthew effect (“the rich get richer”; Hancock et al., 2013). Hancock et al. (2013) modeled three reinforcing psychological processes (cumulative resource allocation, coach expectations, and athlete self-expectations) that widen a modest developmental gap into a durable performance disparity. Wattie et al. (2015) extended this reasoning within a developmental systems framework that frames the RAE as the interaction of individual, task, and environmental constraints, with mitigation possible by altering any one component.

The third mechanism is differential dropout. Children born late in the selection year, having been systematically underselected, are more likely to exit organized sport entirely, not for lack of talent but because early negative experiences erode motivation and self-efficacy (Musch and Grondin, 2001; Delorme et al., 2010). This selective attrition further concentrates the surviving pool toward early-born athletes. Proposed mitigations (rotating cutoffs, expanded bandwidths, bio-banding, corrective adjustments) remain largely theoretical or pilot-stage in practice (Webdale et al., 2020).

Critically, these three mechanisms are difficult to isolate empirically within traditional sports because they operate simultaneously. Physically demanding team sports (the contexts where RAE is strongest) inevitably confound maturation advantages with age-based selection structures and cumulative resource allocation. What is needed to evaluate the mechanistic predictions of the RAE is a competitive domain that systematically differs from traditional sports on one or more of these mechanistic dimensions, thereby functioning as a natural counterfactual.

Esports (organized, competitive video gaming at the professional level) presents precisely such a domain. Three structural features distinguish esports from traditional sports in ways directly relevant to RAE mechanisms. First, physical maturation is largely irrelevant to competitive performance. Success in esports depends on cognitive-motor skills such as reaction time, strategic decision-making, and team coordination rather than on body size, strength, or aerobic capacity (Pluss et al., 2019; Pedraza-Ramirez et al., 2020). Second, formal age-based grouping is absent from the primary developmental pathway. Unlike traditional sports, where youth academies and age-restricted leagues channel talent through annual cohorts, most aspiring esports professionals develop through open online ranking systems (ladders) where opponents are matched by skill rating rather than birthdate (Laxdal and Erikstad, 2025). Players can, in principle, reach professional status at any age without passing through age-gated selection filters. Third, the entry pathway is predominantly self-directed. While team-based esports do involve organizational selection (tryouts, scouting), the initial demonstration of skill occurs in online environments where face-to-face interaction is limited, reducing the social pressures and appearance-based biases that may exaggerate perceived maturation differences in traditional sports.

These structural differences generate clear theoretical predictions. If RAE is primarily driven by physical maturation advantages (Mechanism 1), it should be absent in esports. If it is driven by age-based selection systems (Mechanism 2), it should be absent or greatly attenuated given the lack of age-grouped developmental pathways. If cumulative advantage and dropout effects (Mechanism 3) operate independently of the first two mechanisms, some residual birth quarter variation might persist even in esports. Esports thus provides a rare opportunity to test each mechanism’s contribution in relative isolation.

The relevance of studying RAE in esports extends beyond mechanism decomposition. Two additional features of the esports context make birth-quarter analysis substantively informative independent of any test of traditional RAE mechanisms. First, esports is one of the most competitively intense skill-based domains documented to date, with median professional careers lasting only a few years (Kang, 2025; Kang and Kim, 2025; Thompson et al., 2014) and tournament prize distributions concentrated in a small fraction of competitors (Kang, 2026). In an environment this competitive, even modest differences in early-career timing or developmental advantage could plausibly translate into meaningful career outcomes, making birth-quarter analysis empirically consequential whether or not any specific traditional mechanism applies. Second, esports has undergone rapid institutional formalization over the past decade, with the establishment of national federations, age-restricted youth leagues, formal academies, and the recent inclusion in the Olympic Esports Games (Kang, 2025; Laxdal and Erikstad, 2025). This rapid institutionalization creates a unique natural setting in which traditional RAE patterns may emerge for the first time as governance structures mature, motivating the cohort-level analyses below.

Despite this theoretical promise, empirical evidence remains remarkably thin. Only two studies have examined RAE in esports. Jakobsson et al. (2021), in a multi-sport Swedish study, analyzed 47,030 registered esports athletes alongside participants in physical and cognitive sports. They found a significant RAE among esports players under age 15 (with effect sizes comparable to those in cross-country skiing), but an inverse RAE among adults aged 21–39. Their sample, however, was highly heterogeneous, encompassing all registered players from recreational to elite levels, and did not control for population birth seasonality. Laxdal and Erikstad (2025) conducted the first study focused exclusively on professional esports, collecting birth months for 15,734 elite players across ten game titles from the Liquipedia database. They found no practically meaningful RAE at the professional level (all Cramer’s V < 0.05), a finding highlighted in a subsequent editorial as evidence that esports may develop talent under fairer conditions than traditional sports (Castro et al., 2025).

While these two studies provide an important baseline, they share several limitations that constrain theoretical inference. Neither study controlled for natural seasonal variation in births, a critical omission, given that Northern Hemisphere birth rates peak in summer months (Buckles and Hungerman, 2013; Currie and Schwandt, 2013), which could create the appearance of an inverse RAE when testing against a uniform distribution. Both studies examined only the entry question (“who becomes a professional?”) without analyzing post-entry career trajectories, leaving open whether birth quarter influences career longevity, earnings, or performance conditional on having reached the professional level. Neither study exploited cross-national variation in cutoff dates to test the causal mechanism, despite the availability of esports data spanning dozens of countries with different academic calendars. And neither investigated the pathways through which birth quarter might indirectly influence career outcomes, for instance, through its relationship with debut age, which recent research has identified as a critical determinant of esports career duration and earnings (Kang and Kim, 2025; Kang, 2026).

The broader esports career literature provides important context for the post-entry questions. Cognitive-motor reaction times in StarCraft 2 players have been shown to begin declining around age 24 (Thompson et al., 2014), and large-scale analyses of esports tournament records indicate that competitive performance peaks in the early twenties with relatively short competitive lifespans across many titles (Kang, 2025, 2026). League of Legends specifically shows median professional careers of only a few years, with early specialization associated with shorter careers (Kang, 2025; Kang and Kim, 2025). Previous studies have also documented psychological and vocational challenges associated with transition out of professional esports (Smithies et al., 2020; Zhao et al., 2024). Collectively, this literature establishes that age and timing matter in esports, but the specific contribution of birth quarter has not been investigated beyond the entry stage.

The present study addresses these gaps through four research questions:

*RQ1:* Does a relative age effect exist among professional esports players after controlling for population birth seasonality? If so, does it vary across birth cohorts, debut ages, career durations, and competitive structures?

*RQ2:* Does the academic or sport selection cutoff date causally influence professional esports career outcomes? Specifically, does the same birth month produce different outcomes depending on the player’s country of origin and its corresponding cutoff system?

*RQ3:* Conditional on reaching professional status, does birth quarter influence career longevity or cumulative earnings?

*RQ4:* Does birth quarter operate indirectly through debut age, the age at which a player first enters professional competition, to influence career outcomes?

By examining these questions across 15,065 players from 177 countries competing in 609 game titles, this study leverages the structural uniqueness of esports as a natural counterfactual to evaluate which observable birth-distribution patterns are consistent with each of the three traditional RAE mechanisms. In doing so, it extends the existing two-study evidence base from the entry question to the full career trajectory, introduces population-adjusted and cross-national causal tests not previously applied to esports RAE data, and identifies a previously undocumented mediation pathway through debut age.

## 2 Materials and methods

### 2.1 Data source and sample

This study employed a retrospective, cross-sectional observational design using the Esports Earnings database (esportsearnings.com), a community-curated public repository documenting publicly reported tournament prize awards across global competitive gaming since 1997. Data were extracted between January 2 and January 14, 2026 through systematic retrieval of the database’s publicly accessible player, tournament, game, and country records using official API. The database is maintained by volunteer contributors who compile information from official tournament results, public broadcasts, and verified team announcements, with cross-validation against publisher announcements for major events. At the time of data extraction, the database contained records for 151,432 unique players, 865,608 player–tournament entries, 70,497 tournaments, 609 distinct game titles, and 177 countries. Each player record includes a unique identifier, nickname, date of birth (where publicly known), country of origin, cumulative prize earnings in USD, number of tournament entries, and dates of first and last recorded tournament participation. Each tournament record contains start and end dates, game title, location, prize pool, team versus individual format, and participant counts.

Throughout this paper, the term “professional players” refers to individuals included in the Esports Earnings database, that is, competitors who have received at least one publicly documented prize award in a tournament. This operational definition follows from the scope of the underlying data source. No additional prize-amount cutoff was imposed, because any monetary threshold would conflate skill level with game-specific prize-pool economics (for example, League of Legends prize pools dwarf those of fighting games regardless of competitive intensity). The 15,065 players retained after preprocessing therefore constitute the universe of birthdate-disclosing prize-earning competitors documented in the database during the study period.

Of the 151,432 players in the database, 15,397 (10.2%) had a confirmed date of birth. Players with known birthdates were substantially more prominent than those without: they had higher median career earnings ($7,186 vs. approximately $200 for the 136,035 players without disclosed birthdates), longer careers (mean 3.66 years vs. shorter durations among the no-DOB subset), and more tournament entries (mean 20.3 vs. lower entry counts in the no-DOB subset). This pattern is consistent with the expectation that biographical information is more readily available for higher-profile players who compete in documented, high-stakes events.

Descriptive statistics for the analytical sample are presented in Table 1. Players spanned birth years from 1960 to 2011, with a median birth year of 1998. The mean debut age was 20.3 years (SD = 4.11), and the mean career duration was 3.66 years (SD = 3.59). Cumulative earnings were heavily right-skewed (mean = $80,830; median = $7,186), reflecting the winner-take-most economics typical of esports prize distributions (Kang, 2026).

**TABLE 1:** Descriptive statistics of the analytical sample (*N* = 15,065).

Variable	N	Mean	SD	Median	Min	Max
Career duration (yr)	15,065	3.66	3.59	2.86	0.00	24.97
Debut age (yr)	15,031	20.29	4.11	19.46	10.01	49.88
Total earnings (USD)	15,065	80,830	311,478	7,186	0	12,428,882
Log earnings	15,065	8.79	2.60	8.88	0.00	16.33
Tournament entries	15,065	20.16	36.14	8	1	755
Birth year	15,065	1997.1	7.04	1998	1960	2011

Debut age calculated as (debut date - date of birth)/365.25. Players with debut age < 10 or > 50 excluded from debut age statistics (*N* = 34). Total earnings in USD from all recorded tournaments.

### 2.2 Preprocessing: the January 1st placeholder problem

Initial inspection of birth date distributions revealed a pronounced anomaly: 308 players (2.0% of those with known dates of birth) were recorded as born on January 1st, 7.3 times the expected proportion under a uniform daily distribution (1/365.25 ≈ 0.27%). These January 1st entries differed qualitatively from the rest of the sample across multiple indicators: their median career earnings were $200 (vs. $7,186 for non-January 1st players), mean career duration was 1.57 years (vs. 3.66 years), and mean tournament entries numbered 7.6 (vs. 20.3). Furthermore, January 1st births were disproportionately concentrated among players from countries with less developed esports information infrastructure, notably India (19.6% of Indian players with known dates of birth had January 1st entries), Azerbaijan (21.2%), Turkey (11.8%), and Kazakhstan (12.7%), whereas well-documented esports nations showed much lower rates (South Korea: 0.7%; China: 0.5%).

This pattern strongly suggests that the Esports Earnings database assigns January 1st as a default placeholder when only a player’s birth year, but not the precise month and day, is known, a practice documented in other large-scale sports databases (cf. the use of July 1st defaults in some football databases). Because including these entries would systematically inflate January counts and distort birth quarter analyses, all 308 January 1st births were excluded.

An additional 24 players with implausible birth years (before 1960 or after 2011) were also removed, yielding a main analytical sample of *N* = 15,065.

To ensure robustness against residual date-of-birth coding artifacts, two additional samples were constructed. The *Strict* sample (*N* = 14,598) further excluded all players born on the first day of any month (an additional 467 players), guarding against the possibility that other months also use day-one defaults, albeit at lower rates. The *Conservative* sample (*N* = 13,880) excluded all January-born players entirely, eliminating any residual January contamination at the cost of reducing the analysis to 11 birth months. All primary analyses were conducted on the main sample, with Strict and Conservative results reported as robustness checks.

### 2.3 Variables

#### 2.3.1 Birth quarter and birth half

The primary independent variable was birth quarter (BQ), defined relative to a January 1st cutoff, the standard used by the International Olympic Committee (IOC), FIFA, and most international esports governing bodies. Q1 comprised players born January through March (the “relatively oldest” within a calendar-year cohort), Q2 April through June, Q3 July through September, and Q4 October through December (the “relatively youngest”). Birth half was coded as H1 (January–June) or H2 (July–December) for analyses requiring greater statistical power.

#### 2.3.2 Adjusted birth quarter and relative age

For the cross-national quasi-experiment (RQ2), birth quarters were recalculated relative to each country’s actual academic or sport selection cutoff date. Cutoff months were determined from national education ministry records and sport federation regulations for 20 countries, covering 10,547 players (70.0% of the main sample). Countries were classified into four cutoff groups: January (South Korea, France, Sweden, Finland, Denmark, Norway, Spain, Italy, Australia; *n* = 9), September (China, United Kingdom, Russia, Ukraine, United States, Canada, Poland; *n* = 7), April (Japan; *n* = 1), and other months (Brazil: March; Germany: July; Netherlands: October; *n* = 3). The adjusted birth quarter reassigned Q1 through Q4 relative to each country’s cutoff, such that adjusted Q1 always represented the “relatively oldest” group within that country’s system. South Korea is classified under the current formal January 1 cutoff (effective from the 2009 academic year reform); the Korean Illustration (section 3.2.3) reports parallel analyses under both the January and the pre-2009 March cutoffs to assess sensitivity to this classification.

A continuous relative age variable (ranging from 0 to 1) was computed as the normalized distance from the cutoff month, where values approaching 1 indicated birth immediately after the cutoff (oldest in cohort) and values approaching 0 indicated birth just before the cutoff (youngest in cohort).

#### 2.3.3 Debut age and career duration

Debut age was calculated as the difference between a player’s first recorded tournament date and their date of birth, expressed in years (divided by 365.25). Players with computed debut ages below 10 or above 50 years were flagged as invalid (*n* = 34) and excluded from debut-age-specific analyses. Career duration was taken directly from the database as the span between first and last tournament entries, in years.

#### 2.3.4 Career outcomes

Two outcome variables captured career success. Log earnings was computed as the natural logarithm of cumulative USD prize earnings plus one, i.e., log(1 + earnings), to address the extreme right skew in the raw earnings distribution. Tournament count recorded the total number of tournament entries per player.

#### 2.3.5 Event indicator for survival analysis

For career longevity analyses, a binary event indicator was constructed. A player was classified as “retired” (event = 1) if their last recorded tournament participation preceded the most recent date in the database by two or more years. Players whose last entry fell within the two-year window were treated as right-censored (event = 0). Sensitivity analyses using one-year and three-year thresholds were conducted to assess robustness to this definition.

#### 2.3.6 Team play

Players were classified as predominantly team-based (is_team = 1) if 50% or more of their tournament entries were in team-format competitions (Teamplay = 1 in the tournament records), and as predominantly individual (is_team = 0) otherwise.

### 2.4 Statistical analysis

#### 2.4.1 RQ1: prevalence of RAE

The presence of RAE was assessed using chi-square (χ^2^) goodness-of-fit tests comparing the observed birth quarter distribution against two reference distributions. First, a uniform distribution (expected frequency = N/4 per quarter) served as the standard benchmark used in most RAE studies (Cobley et al., 2009). Second, a population-adjusted distribution was used to account for natural seasonal variation in birth rates. Because the sample spanned 177 countries, country-specific monthly birth statistics were not available for all nations. As a pragmatic solution, the average of United States (Centers for Disease Control and Prevention, and National Center for Health Statistics, 2015–2019) and South Korean (Statistics Korea, 2000–2010) monthly birth proportions were used as a proxy for the global esports population, which is concentrated in North America and East Asia. This two-country average was normalized to sum to 1.0 and used to generate expected birth counts per month and per quarter. The population-adjusted test is critical because the Northern Hemisphere exhibits well-documented birth seasonality, with births peaking in July through September (Buckles and Hungerman, 2013; Currie and Schwandt, 2013), which could create the appearance of an inverse RAE (overrepresentation of Q3–Q4) when none exists.

Effect sizes were quantified using Cramer’s V, with established thresholds of < 0.10 (negligible), 0.10–0.30 (small), 0.30–0.50 (medium), and > 0.50 (large) (Cohen, 1988). Odds ratios (OR) comparing Q1 to Q4 representation were computed with 95% Wald confidence intervals. An OR significantly below 1.0 would indicate an inverse RAE (Q4 overrepresentation); an OR significantly above 1.0 would indicate a traditional RAE (Q1 overrepresentation).

Subgroup analyses were conducted by birth cohort (six groups: ≤ 1985, 1986–1990, 1991–1995, 1996–2000, 2001–2005, 2006+), debut age (five groups: < 16, 16–18, 18–21, 21–25, > 25), career duration (five groups: < 1, 1–3, 3–5, 5–10, > 10 years), and team versus individual play. Given the number of subgroup comparisons, Benjamini–Hochberg false discovery rate (FDR) correction was applied, with uncorrected *p*-values also reported.

#### 2.4.2 RQ2: causal effect of academic cutoffs

To test whether the academic or sport selection cutoff causally influences esports career outcomes (the central mechanism driving RAE in traditional sports; Musch and Hay, 1999; Helsen et al., 2005), cross-national variation in cutoff dates was exploited as a quasi-experimental design. If cutoff-based maturation advantages drive birth quarter effects, the same birth month should yield different outcomes depending on a player’s country of origin (and thus their position relative to the local cutoff).

Three analytical approaches were employed. First, chi-square tests were conducted within each country using adjusted birth quarters, with pooled tests by cutoff group (January vs. September). Second, ordinary least squares (OLS) regression estimated the effect of relative age on log earnings with country fixed effects, thereby controlling for all time-invariant between-country differences:

log(earnings) = β_0_ + β_1_ ⋅ relative_age + Σβ_c ⋅ country_c + β_2_ ⋅ debut_age + Σβ_g ⋅ genre_g + ε

Third, an interaction model tested whether the relationship between birth quarter and earnings differed across cutoff groups:

log(earnings) = β_0_ + β_1_ ⋅ cutoff_group + β_2_ ⋅ BQ + β_3_ ⋅ (cutoff_group × BQ) + Σβ_g ⋅ genre_g + ε

A joint F-test of the interaction terms assessed whether cutoff group moderated the BQ–earnings relationship. A non-significant interaction would indicate that the same birth month produces equivalent outcomes regardless of its relative position within different national cutoff systems.

As an illustrative case, South Korea’s 2009 shift from a March to January academic cutoff was examined. The same 1,216 Korean players were analyzed under both cutoff definitions to demonstrate the sensitivity (or insensitivity) of RAE conclusions to cutoff choice.

#### 2.4.3 RQ3: post-entry career trajectory effects

Career longevity was analyzed using Kaplan–Meier survival estimation (Kaplan and Meier, 1958) and Cox proportional hazards regression (Cox, 1972). The multivariate log-rank test compared survival distributions across the four birth quarters, with pairwise Q1 versus Q4 and H1 versus H2 comparisons. Two Cox models were fitted: Model 1 included only birth quarter dummies (reference: Q1), and Model 2 additionally controlled for debut age, team play, and game genre (top five genres as dummies). The proportional hazards assumption was assessed via Schoenfeld residual tests. The concordance index (C-statistic) was reported as a measure of model discrimination.

Earnings differences were assessed via Kruskal–Wallis tests (given the non-normal distribution) and OLS regression of log earnings on birth quarter with controls. To isolate the causal effect of birth quarter from potential confounders, propensity score matching (PSM) was employed (Rosenbaum and Rubin, 1983; Austin, 2011). A logistic regression model estimated the propensity of being born in Q1 (vs. Q4) as a function of game genre, country of origin (top 10 countries; remainder grouped as “other”), debut age, and debut year. One-to-one nearest-neighbor matching with a caliper of 0.05 standard deviations of the propensity score was applied. Covariate balance was assessed using standardized mean differences (SMD), with | SMD| < 0.10 indicating acceptable balance. Post-matching outcome differences were tested using Mann–Whitney U tests.

#### 2.4.4 RQ4: debut age mediation

The indirect pathway from birth quarter through debut age to career earnings was estimated through three regression models:

First, the total effect (path c) of BQ_late on log earnings was estimated as log_earnings = β_0_ + c ⋅ BQ_late + controls + ε.

Second, path a was estimated by regressing debut age on BQ_late: debut_age = β_0_ + a ⋅ BQ_late + controls + ε.

Third, the mediator was added to the outcome model to estimate path b and the residual direct effect (c’) simultaneously: log_earnings = β*0* + c’ ⋅ BQ_late + b ⋅ debut_age + controls + ε.

where BQ_late was coded as 1 for players born in Q3 or Q4 (July–December) and 0 for Q1 or Q2, and controls included game genre dummies and team play. Mediation was assessed using the conventional Baron and Kenny (1986) causal-steps logic supplemented by the Sobel (1982) test of the indirect effect (Preacher and Hayes, 2004): (a) the total effect c was estimated and reported, (b) BQ_late was tested for its association with debut age (path a), (c) debut age was tested for its association with earnings controlling for BQ_late (path b), and (d) the direct effect c’ was compared with the total effect c to characterize attenuation. The indirect effect (a × b) was tested using the Sobel test statistic, with a significant indirect effect interpreted as the primary quantitative evidence of mediation. The percentage mediated is reported descriptively as (a × b)/c × 100.

To examine whether the mediation pathway differed by competitive structure, subgroup analyses were conducted separately for team-based and individual players. Additionally, a two-way interaction between birth quarter and debut age group (early: < 18, mid: 18–22, late: > 22) was tested in predicting log earnings, with a joint F-test assessing the significance of the interaction terms.

### 2.5 Software and reproducibility

All analyses were conducted in Python 3.12 using pandas (v2.2) for data management, SciPy (v1.13) for chi-square and non-parametric tests, statsmodels (v0.14) for OLS and logistic regression, lifelines (v0.29) for survival analysis, and scikit-learn (v1.5) for propensity score estimation. Figures were produced using Matplotlib (v3.9). The complete analytical pipeline, including the preprocessing and analysis scripts for all research questions, the preprocessed analytical samples (main, strict, and conservative), and a verification report reproducing all reported statistics, is provided in the Supplementary material.

## 3 Results

### 3.1 RQ1: is there a relative age effect in professional esports?

#### 3.1.1 Aggregate birth quarter distribution

**FIGURE 1:**
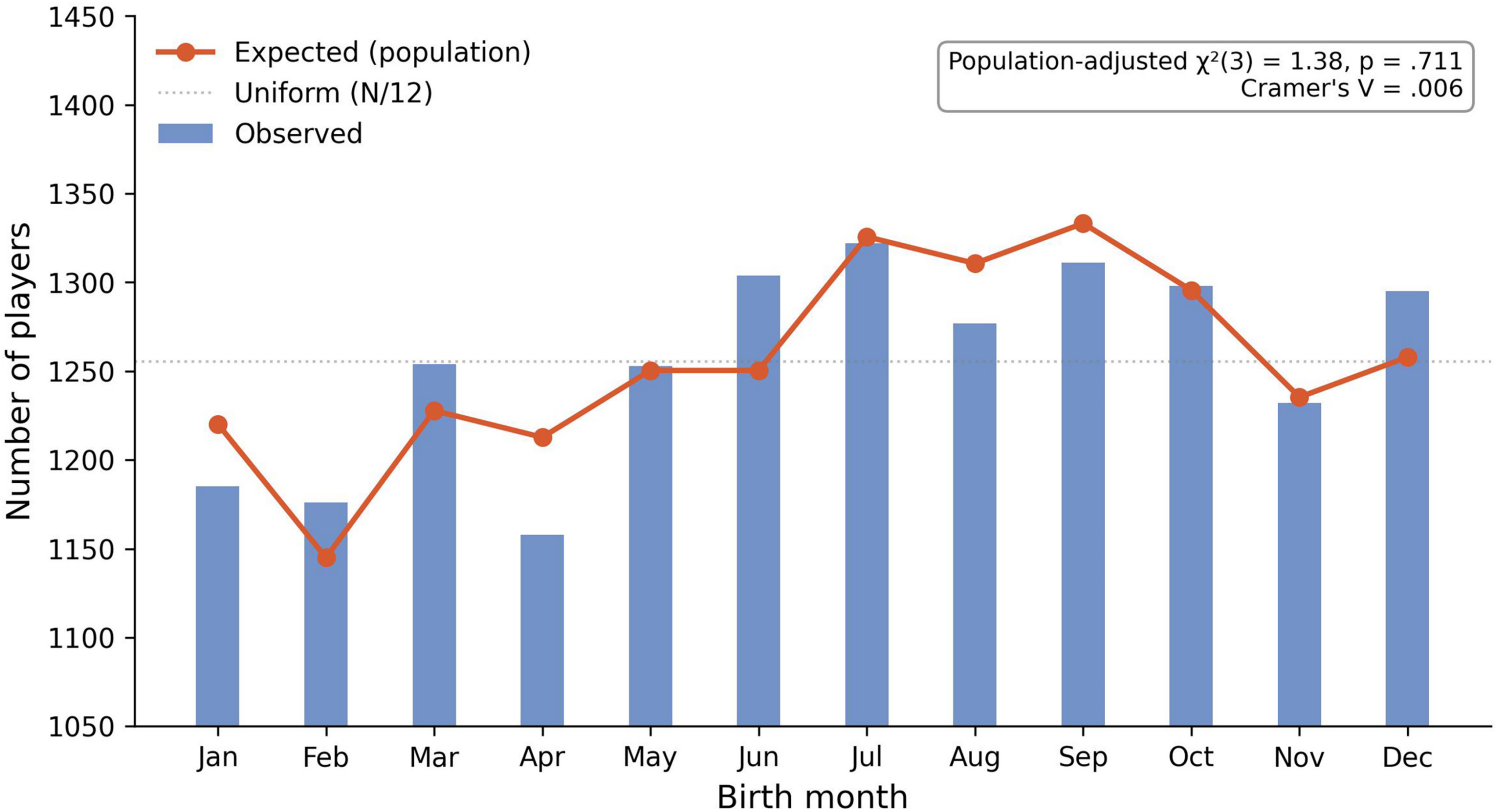
Monthly birth distribution of professional esports players (*N* = 15,065). Blue bars represent observed counts; the orange line represents expected counts based on average U.S. and South Korean monthly birth proportions; the gray dotted line indicates the uniform expectation (N/12). The population-adjusted chi-square test was non-significant [χ^2^(3) = 1.38, *p* = 0.711, Cramer’s V = 0.006], indicating that the observed distribution does not deviate from population birth seasonality.

The monthly birth distribution of the main analytical sample is presented in Figure 1, alongside two reference distributions: a uniform expectation (N/12 per month) and population-adjusted expected counts derived from the average of U.S. and South Korean monthly birth proportions. Visual inspection reveals that the observed distribution closely tracks the population reference, with both exhibiting the characteristic Northern Hemisphere pattern of higher birth counts in summer months (June through September) and lower counts in winter months (January through February).

When tested against a uniform distribution, the birth quarter frequencies [Q1 = 3,615 (24.0%), Q2 = 3,715 (24.7%), Q3 = 3,910 (26.0%), Q4 = 3,825 (25.4%)] reached statistical significance, χ^2^(3) = 13.18, *p* = 0.004, Cramer’s V = 0.017 (Table 2). The direction was inverse to the traditional RAE pattern: Q1 (the “relatively oldest” under a January cutoff) was the least represented quarter, while Q3 was the most represented. The Q1-versus-Q4 odds ratio was 0.928 [95% CI (0.880, 0.978)].

**TABLE 2:** Overall relative age effect tests across analytical samples.

Sample	*N*	Q1%	Q2%	Q3%	Q4%	χ ^2^(3)	*p*	*V*	OR [95% CI]
Uniform reference									
Main	15,065	24.0	24.7	26.0	25.4	13.18	0.004	0.017	0.928 [0.880–0.978]
Strict	14,598	24.3	24.5	25.9	25.3	9.31	0.025	0.015	0.949 [0.899–1.000]
Population-adjusted									
Main	15,065	n.a.	n.a.	n.a.	n.a.	1.38	0.711	0.006	n.a.

Results are presented for both uniform (25% per quarter) and population-adjusted expected frequencies. OR = Odds Ratio (Q1 vs. Q4); V = Cramer’s V. The Strict sample additionally excludes all first-of-month birthdays (*N* = 14,598). OR = Odds Ratio (Q1 vs. Q4). V = Cramer’s V. Population-adjusted analysis uses US + KR average monthly birth proportions as expected frequencies. Strict sample excludes all 1st-of-month birthdays. n.a. indicates not applicable.

However, when expected frequencies were adjusted to reflect population birth seasonality, the effect was no longer statistically detectable: χ^2^(3) = 1.38, *p* = 0.711, Cramer’s V = 0.006 (Table 2). The observed-to-expected ratios for each quarter were Q1 = 1.006, Q2 = 1.000, Q3 = 0.985, and Q4 = 1.010, indicating near-perfect alignment with population base rates. The birth half comparison told a consistent story: H2 (51.3%) exceeded H1 (48.7%), χ^2^(1) = 10.89, *p* = 0.001, but this difference is fully attributable to the well-documented summer birth peak rather than to any selection mechanism. Results from the Strict sample (*N* = 14,598) confirmed the same pattern: the uniform-reference test remained marginally significant [χ^2^(3) = 9.31, *p* = 0.025, *V* = 0.015], but the effect was negligible in magnitude.

#### 3.1.2 Cohort-specific reversal pattern

**FIGURE 2:**
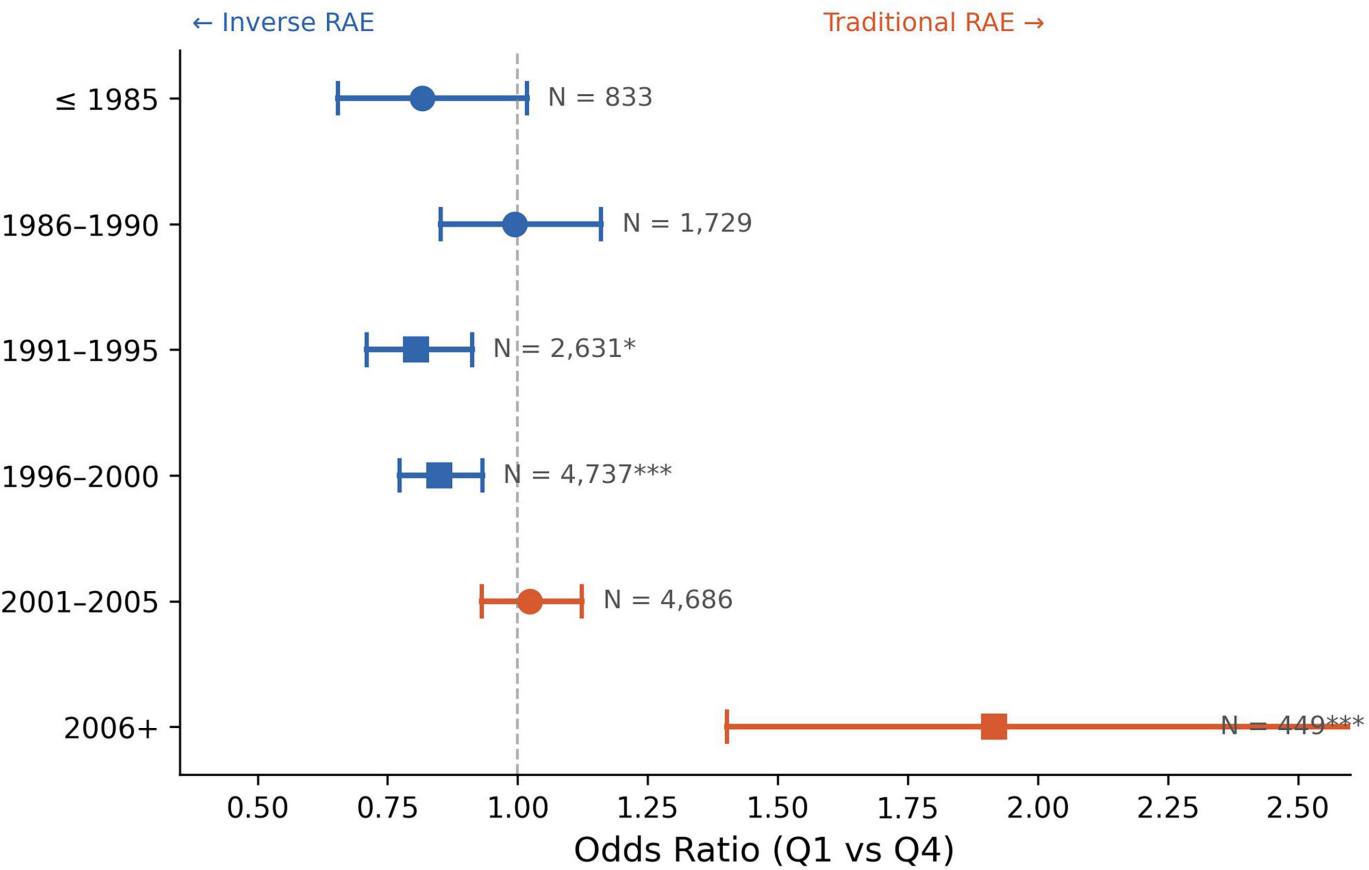
Cohort-specific odds ratios (Q1 vs. Q4) with 95% confidence intervals. Blue markers indicate cohorts with inverse RAE direction (OR < 1.0); orange markers indicate cohorts with traditional RAE direction (OR > 1.0). Filled squares denote statistically significant effects (*p* < 0.05); filled circles denote non-significant effects. The dashed vertical line marks OR = 1.0 (no effect). The 2006+ cohort shows a pronounced traditional RAE (OR = 1.92, *p* < 0.001), while the 1991–2000 cohorts show significant inverse RAE (OR = 0.81–0.85).

Although the aggregate picture was null, subgroup analyses revealed a striking and internally consistent pattern across birth cohorts. Table 3 presents the full results; Figure 2 displays the cohort-specific odds ratios with 95% confidence intervals.

**TABLE 3:** Subgroup analyses of birth quarter distribution by birth cohort, debut age, career duration, and competitive structure (team vs. individual).

Subgroup	*N*	Q1%	Q4%	OR (Q1/Q4)	χ(3)	*p*	*V*
Birth cohort							
≤ 1985	833	23.5	27.4	0.816	2.79	0.425	0.033
1986–1990	1,729	24.5	24.6	0.994	0.83	0.843	0.013
1991–1995	2,631	22.8	26.8	0.805	9.02	0.029*	0.034
1996–2000	4,737	23.0	26.0	0.849	19.05	< 0.001***	0.037
2001–2005	4,686	25.0	24.6	1.023	1.70	0.637	0.011
2006+	449	30.3	18.5	1.916	16.79	< 0.001***	0.112
Debut age							
< 16	1,085	21.4	28.4	0.686	10.98	0.012*	0.058
16–18	3,236	23.3	25.8	0.872	10.82	0.013*	0.033
18–21	5,917	23.4	25.5	0.893	13.32	0.004**	0.027
21–25	3,277	24.9	24.7	1.007	0.20	0.977	0.005
> 25	1,516	27.8	23.5	1.257	6.81	0.078	0.039
Career duration							
< 1 year	4,012	25.5	24.6	1.052	1.02	0.796	0.009
1–3 years	3,756	23.1	26.0	0.851	9.14	0.028*	0.029
3–5 years	3,117	25.1	25.2	0.995	3.20	0.361	0.019
5–10 years	3,254	21.8	26.2	0.788	18.82	< 0.001***	0.044
> 10 years	926	25.3	24.4	1.047	0.32	0.957	0.011
Team vs. Individual							
Team ( ≥ 50%)	12,294	23.5	25.5	0.895	23.69	< 0.001***	0.025
Individual ( < 50%)	2,771	26.4	24.9	1.085	3.64	0.303	0.021

OR = Q1/Q4 odds ratio. All tests are against a uniform (25%) expected distribution. OR = Q1/Q4 odds ratio. Tests against uniform (25%) expected distribution.

*p < 0.05,

**p < 0.01,

***p < 0.001.

Among players born in 1991–1995 (*N* = 2,631), Q1 was underrepresented at 22.8% versus Q4 at 26.8%, yielding an OR of 0.805 (*p* = 0.029). This inverse pattern was even more pronounced in the 1996–2000 cohort (*N* = 4,737): Q1 = 23.0%, Q4 = 26.0%, OR = 0.849 (*p* < 0.001). In sharp contrast, the youngest cohort, players born in 2006 or later (*N* = 449, mean debut age 15.7 years), exhibited a strong traditional RAE: Q1 = 30.3%, Q4 = 18.5%, OR = 1.916 (*p* < 0.001). The intermediate cohorts showed transitional patterns: the 2001–2005 cohort (*N* = 4,686) was near-uniform (OR = 1.023, *p* = 0.637), and pre-1990 cohorts showed non-significant inverse trends.

#### 3.1.3 Debut age gradient

**FIGURE 3:**
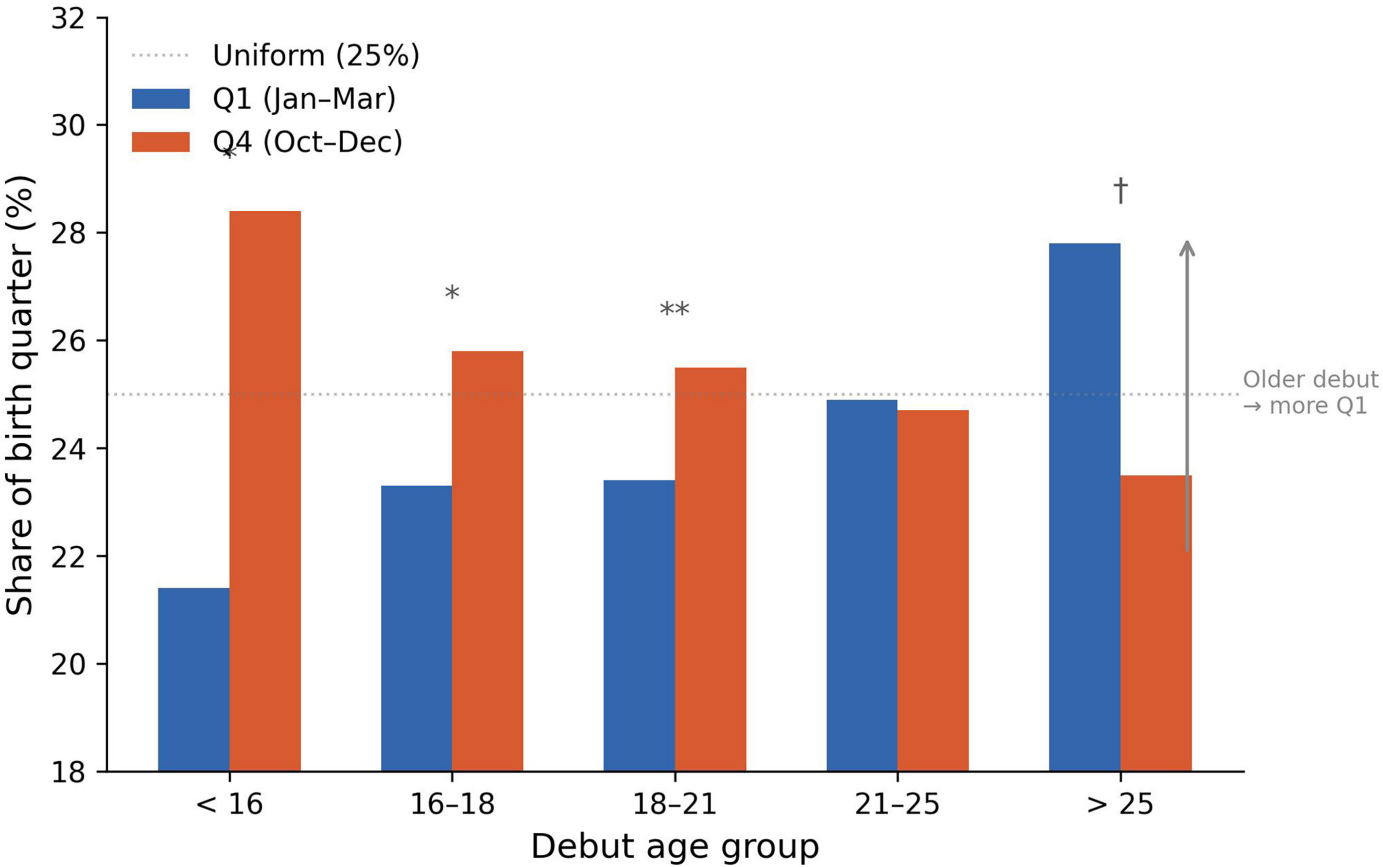
Birth quarter representation by debut age group. Blue bars represent Q1 (January–March) share; orange bars represent Q4 (October–December) share. The gray dotted line indicates the uniform expectation (25%). A clear linear gradient is visible: Q4 is overrepresented among early-debut players ( < 16 years) and Q1 is overrepresented among late-debut players ( > 25 years), with the crossover occurring around ages 21–25. Significance markers: *p < 0.05, **p < 0.01, ^†^*p* < 0.10.

The cohort pattern was mirrored by a continuous gradient across debut age groups (Figure 3 and Table 3). Among players who debuted before age 16 (*N* = 1,085), Q4 was markedly overrepresented (28.4%) relative to Q1 (21.4%), yielding a Q1–Q4 difference of -7.0 percentage points [χ^2^(3) = 11.03, *p* = 0.012, *V* = 0.058]. This inverse pattern attenuated progressively through the 16–18 (Q1–Q4 = -2.5 pp, *p* = 0.012) and 18–21 groups (-2.1 pp, *p* = 0.004), crossed the equilibrium point in the 21–25 group (Q1 = 24.9%, Q4 = 24.7%, *p* = 0.977), and reversed in the over-25 group (Q1 = 27.8%, Q4 = 23.5%, +4.4 pp, *p* = 0.078). The Spearman correlation between birth quarter and debut age was ρ = –0.025 (*p* = 0.003), and a logistic regression confirmed that each additional year of debut age increased the probability of being Q1-born by 1.5% (OR = 1.015, *p* = 0.001).

#### 3.1.4 Additional subgroup patterns

Two further subgroup patterns appear in Table 3. The inverse RAE was most pronounced among long-career players: those with 5–10 years of career duration showed Q1 = 21.8% and Q4 = 26.2% (OR = 0.788, *p* < 0.001), while players with careers under 1 year showed a near-uniform distribution (*p* = 0.796). The pattern also differed by competitive structure: team-based players exhibited a significant inverse RAE (OR = 0.895, *p* < 0.001), whereas individual players showed a non-significant tendency in the traditional direction (OR = 1.085, *p* = 0.303).

### 3.2 RQ2: does the academic cutoff causally affect esports careers?

#### 3.2.1 Country-level adjusted birth quarter tests

**FIGURE 4:**
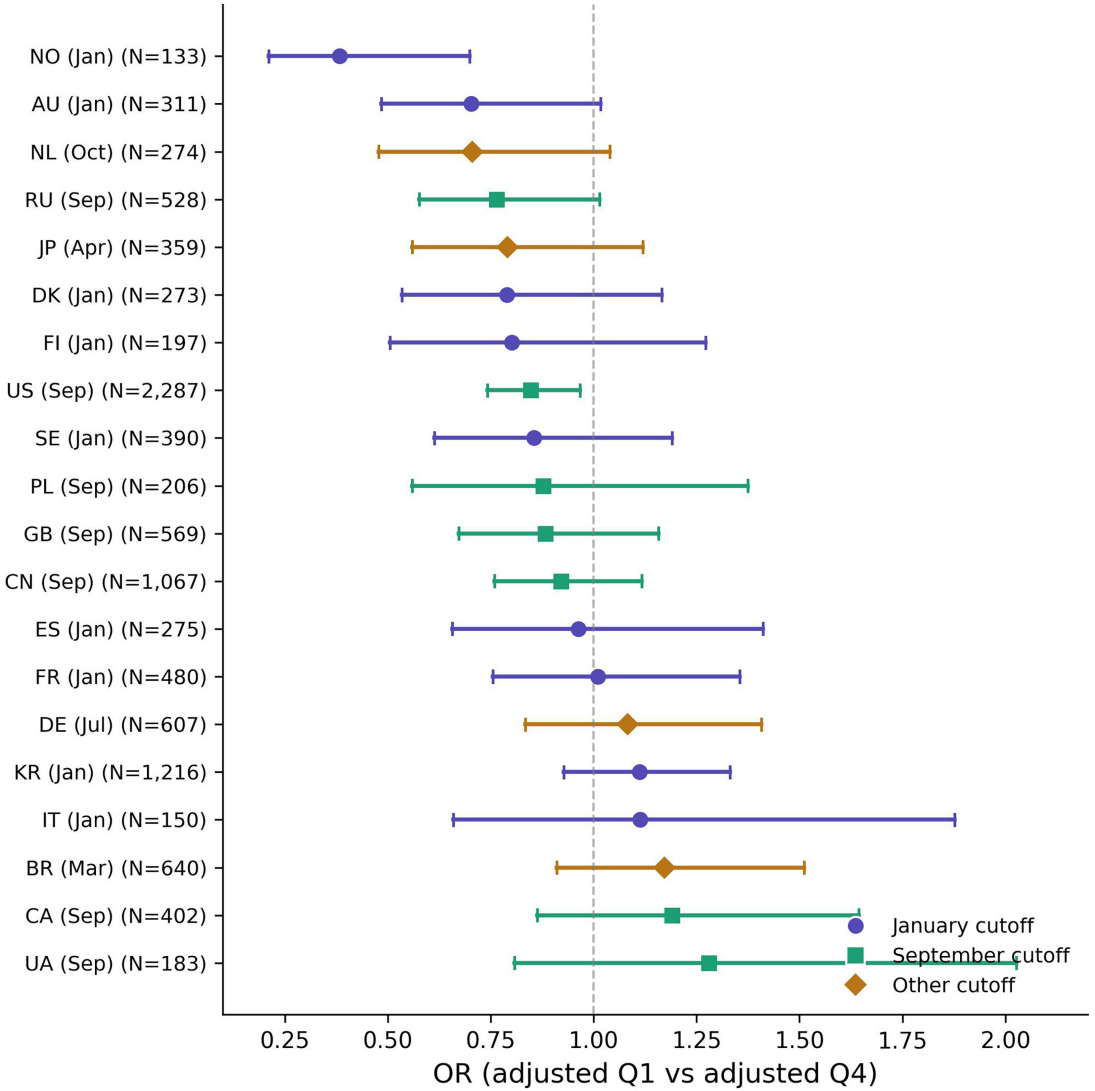
Country-level adjusted birth quarter odds ratios (adjusted Q1 vs. adjusted Q4) with 95% confidence intervals for 20 countries with N ≥ 100. Birth quarters were realigned to each country’s academic or sport selection cutoff date. Purple circles indicate January-cutoff countries; green squares indicate September-cutoff countries; gold diamonds indicate countries with other cutoff months. Countries are ordered by ascending odds ratio.

Table 4 presents the results of RAE tests for the 11 largest country cohorts (each *N* ≥ 350), using adjusted birth quarters aligned to each nation’s academic cutoff. The full set of 20 classified countries (all with *N* ≥ 100) is shown in Figure 4. The results were heterogeneous. The United States (September cutoff; *N* = 2,287) showed a significant inverse RAE on the adjusted scale (adjQ1 = 24.3%, adjQ4 = 27.5%, OR = 0.847, *p* < 0.001), as did Norway (January cutoff; *N* = 133, OR = 0.383, *p* = 0.048), though the latter should be interpreted cautiously given the small sample. South Korea (January cutoff; *N* = 1,216) showed a significant but traditional-direction effect (adjQ1 = 27.5%, adjQ4 = 25.5%, OR = 1.111, *p* = 0.027). The remaining countries did not reach significance.

**TABLE 4:** Country-level relative age effect tests with adjusted birth quarters for the 11 largest country cohorts (each *N* ≥ 350).

Country	Cutoff	*N*	adjQ1%	adjQ4%	OR [95% CI]	χ ^2^(3)	*p*	*V*
US	Sep	2,287	24.3	27.5	0.847 [0.742–0.967]	17.13	< 0.001***	0.050
KR	Jan	1,216	27.5	25.5	1.111 [0.928–1.331]	9.16	0.027*	0.050
CN	Sep	1,067	25.4	27.0	0.921 [0.759–1.117]	4.06	0.255	0.036
BR	Mar	640	26.4	23.4	1.172 [0.910–1.511]	1.29	0.732	0.026
DE	Jul	607	25.4	23.9	1.083 [0.834–1.407]	0.52	0.915	0.017
GB	Sep	569	23.0	25.3	0.883 [0.673–1.158]	1.45	0.693	0.029
RU	Sep	528	21.8	26.7	0.764 [0.576–1.014]	3.11	0.376	0.044
FR	Jan	480	25.0	24.8	1.011 [0.755–1.355]	3.52	0.319	0.049
CA	Sep	402	26.1	22.9	1.191 [0.863–1.644]	4.11	0.250	0.058
SE	Jan	390	22.1	24.9	0.855 [0.613–1.191]	3.25	0.355	0.053
JP	Apr	359	21.2	25.3	0.791 [0.559–1.119]	3.08	0.379	0.054
Pooled results								
Jan group	Jan	3,425	24.6	25.9	0.931	3.11	0.541	0.017
Sep group	Sep	5,378	24.4	26.8	0.882	12.76	0.003**	0.028

Birth quarters were realigned to each country’s academic or sport selection cutoff date. Pooled results aggregate countries within each cutoff group. OR = adjusted Q1 vs. adjusted Q4 odds ratio. Adjusted BQ realigns birth quarters to each country’s academic/sport cutoff date. Jan, January 1; Sep, September 1, etc. Pooled results aggregate all countries within each cutoff group.

*p < 0.05,

**p < 0.01,

***p < 0.001.

When pooled by cutoff group, September-cutoff countries (*N* = 5,378) showed a significant inverse RAE (OR = 0.882, *p* = 0.003), while January-cutoff countries (*N* = 3,425) did not (OR = 0.931, *p* = 0.541). April-cutoff (Japan, *N* = 359) and March-cutoff (Brazil, *N* = 640) groups were also non-significant. Germany (July cutoff, *N* = 607) and Netherlands (October cutoff, *N* = 274) are presented at the country level in Figure 4 but are not pooled separately, given that each constitutes a unique cutoff month within the sample.

#### 3.2.2 Causal inference tests: consistent null

Despite the country-level variation in adjusted birth quarter distributions, all tests designed to isolate a causal cutoff effect returned null results. The OLS regression of log earnings on relative age with country fixed effects, which controls for all stable between-country differences and thereby isolates the within-country effect of cutoff position, yielded a near-zero coefficient: β = +0.004 (*p* = 0.959), unchanged when controls for game genre and debut age were added. The Kruskal–Wallis test comparing earnings across adjusted birth quarters was also non-significant (*H* = 3.56, *p* = 0.313), as was the test for career duration (*H* = 0.46, *p* = 0.928). The Spearman correlation between relative age and log earnings was ρ = +0.008 (*p* = 0.403).

The interaction model testing whether cutoff group (January vs. September) moderated the relationship between birth quarter and earnings produced entirely non-significant interaction terms (all *p* > 0.61), with a joint *F*-test of *p* = 0.886. This indicates that the same birth month produces statistically indistinguishable career outcomes regardless of whether it falls in Q1 or Q3 of the local cutoff system.

#### 3.2.3 The Korean illustration

The same 1,216 Korean players were analyzed under both cutoffs. Under the March cutoff (the system in effect for their academic cohort, as all had birth years before 2009), the result was adjQ1 = 22.6%, adjQ4 = 27.1%, OR = 0.788, *p* = 0.022. Under a January cutoff, the result was adjQ1 = 27.5%, adjQ4 = 25.5%, OR = 1.111, *p* = 0.027.

### 3.3 RQ3: do birth quarter effects persist post-entry?

#### 3.3.1 Career longevity

**FIGURE 5:**
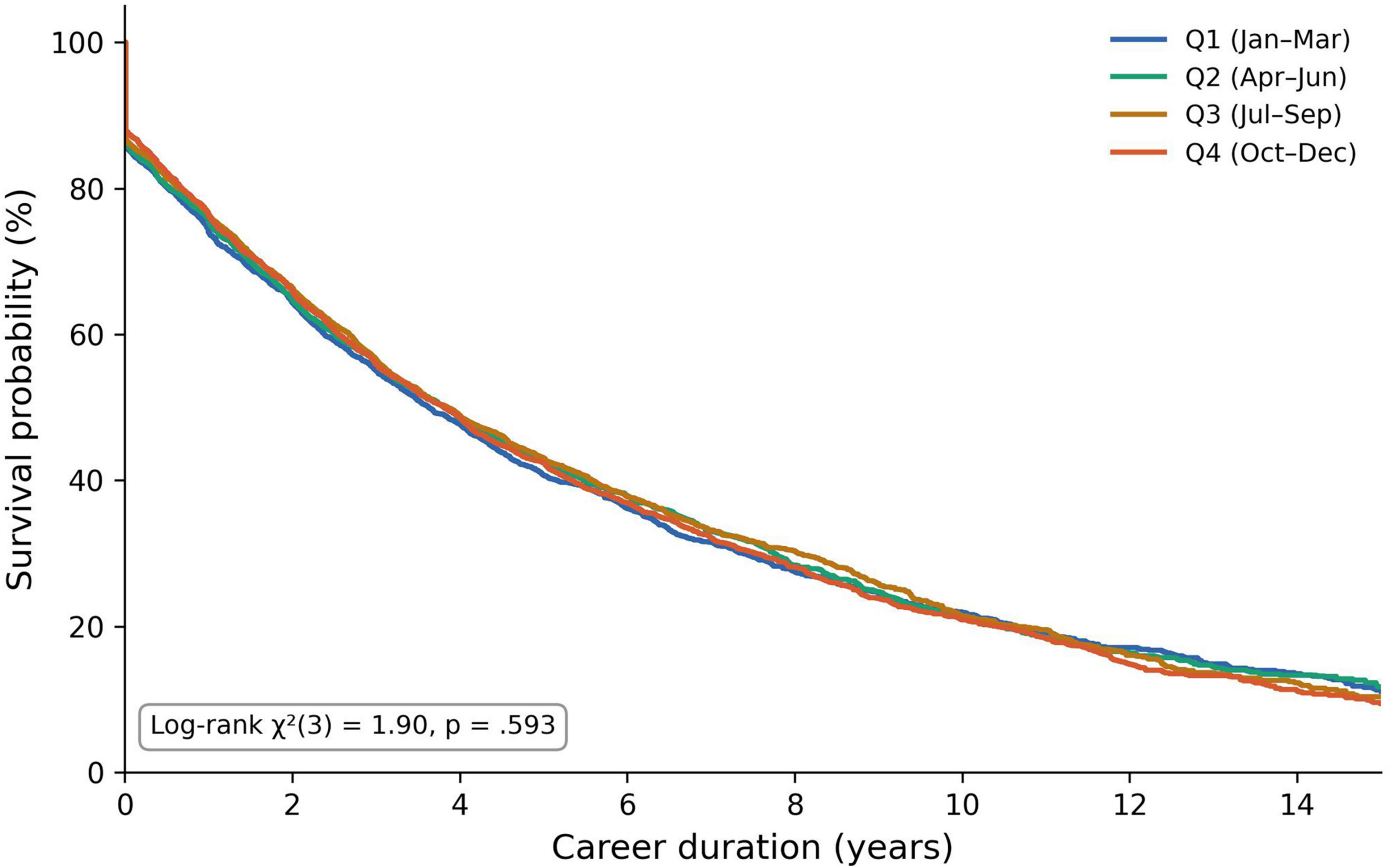
Kaplan–Meier survival curves by birth quarter (*N* = 15,065). Retirement was defined as two or more years since the last recorded tournament entry. The four curves are virtually superimposed, and the multivariate log-rank test was non-significant [χ^2^(3) = 1.90, *p* = 0.593)], indicating no birth quarter difference in career longevity. Median survival times: Q1 = 3.63 years, Q2 = 3.81, Q3 = 3.85, Q4 = 3.81.

Kaplan–Meier survival curves for the four birth quarters are presented in Figure 5. The curves were virtually superimposed across the full 15-year observation window. Median survival times were Q1 = 3.63 years, Q2 = 3.81, Q3 = 3.85, and Q4 = 3.81. The multivariate log-rank test was non-significant: χ^2^(3) = 1.90, *p* = 0.593. Pairwise comparisons were also null [Q1 vs. Q4: χ^2^(1) = 0.22, *p* = 0.641; H1 vs. H2: χ^2^(1) = 0.35, *p* = 0.552].

Cox proportional hazards models are summarized in Table 5. In Model 1 (birth quarter only), all hazard ratios were close to unity (Q2: HR = 0.971, *p* = 0.34; Q3: HR = 0.961, *p* = 0.20; Q4: HR = 0.985, *p* = 0.62), and the concordance index was C = 0.506. Model 2 added debut age, team play, and game genre as controls. The birth quarter hazard ratios remained non-significant across all quarters relative to Q1 (Q4 vs. Q1: HR = 1.013, *p* = 0.657; Q2 and Q3 hazard ratios are reported in Table 5 and similarly non-significant). Debut age was a strong predictor: each additional year of debut age was associated with a 9.9% higher retirement hazard (HR = 1.099, 95% CI [1.089, 1.110], *p* < 0.001). Team play was also significant (HR = 1.192, *p* < 0.001). Model 2 achieved C = 0.648. The Schoenfeld residual test was non-significant for all covariates.

**TABLE 5:** Cox proportional hazards models for career duration.

Variable	β	SE	HR	95% CI	*p*
Model 1: BQ only					
Q2 (ref = Q1)	–0.030	0.031	0.971	0.914–1.031	0.340
Q3	–0.040	0.030	0.961	0.906–1.020	0.196
Q4	–0.015	0.031	0.985	0.928–1.046	0.624
Model 2: BQ + controls					
Q2 (ref = Q1)	–0.018	0.031	0.982	0.925–1.043	0.533
Q3	–0.009	0.030	0.992	0.935–1.051	0.766
Q4	+0.013	0.031	1.013	0.954–1.075	0.657
Debut age	+0.095	0.005	1.099	1.089–1.110	< 0.001***
Team play	+0.176	0.029	1.192	1.127–1.262	< 0.001***

Model 1 includes birth quarter only (reference: Q1). Model 2 adds debut age, team play, and game genre controls (genre coefficients not shown). HR, Hazard Ratio; C, concordance index. Event defined as ≥ 2 years since last tournament entry (*N* = 15,031 with valid debut age). HR, hazard ratio; C, Concordance index. Event defined as ≥ 2 years since last tournament entry. Model 2 additionally controls for top 5 game genres (coefficients not shown). Reference category for BQ is Q1 (January–March). *N* = 15,031 (debut age valid).

***p < 0.001.

#### 3.3.2 Earnings

Median earnings by quarter were Q1 = $6,667, Q2 = $7,142, Q3 = $7,642, and Q4 = $7,523. The Kruskal–Wallis test was marginally significant (*H* = 7.59, *p* = 0.055), and the pairwise Mann–Whitney U test comparing Q1 with Q4 reached significance (*p* = 0.015). The effect size was ε^2^ = 0.0003, indicating that birth quarter explained approximately 0.03% of the variance in earnings, far below the conventional benchmark for a small effect (ε^2^ ≈ 0.01).

OLS regression of log earnings on birth quarter with controls for debut age, game genre, and team play yielded non-significant birth quarter coefficients (Q2: β = +0.063, *p* = 0.258; Q3: β = +0.066, *p* = 0.229; Q4: β = +0.066, *p* = 0.229; *R*^2^ = 0.176). A joint *F*-test for birth month dummies (11 df), after controlling for debut age, genre, and team play, was *F* = 1.12, *p* = 0.343.

#### 3.3.3 Propensity score matching

To further isolate the birth quarter effect from compositional differences, PSM was conducted comparing Q1 (*N* = 3,606) and Q4 (*N* = 3,818) players. After matching on game genre, country, debut age, and debut year, 3,606 pairs were retained within the 0.05 caliper (Table 6). Covariate balance was satisfactory: standardized mean differences for debut age (SMD = +0.073) and debut year (SMD = –0.066) were both below the conventional 0.10 threshold. The Q1 and Q4 analytical Ns reported here (3,606 and 3,818) are slightly smaller than the corresponding aggregate counts (3,615 and 3,825) because the PSM logistic regression required complete data on all covariates; 9 Q1 and 7 Q4 players were excluded due to missing values on game genre, country (top-10 grouping), debut age, or debut year.

**TABLE 6:** Propensity score matching: covariate balance and outcome comparison for Q1 vs. Q4 players.

Variable	Q1 (matched)	Q4 (matched)	SMD/Δ	*P*
Balance check				
Debut age (yr)	20.48	20.18	+0.073	n.a.
Debut year	2017.27	2017.61	–0.066	n.a.
Outcome comparison				
Earnings (median $)	6,668	8,217		0.487 0.487 0.820
Log earnings (median)	8.80	9.01		
Duration (median yr)	2.80	3.10		

Propensity scores were estimated from game genre, country (top 10), debut age, and debut year via logistic regression. Matching: 1:1 nearest-neighbor with caliper = 0.05 SD. SMD = Standardized Mean Difference (| SMD| < 0.10 indicates acceptable balance). Outcome *p*-values from Mann–Whitney U tests. PSM via 1:1 nearest-neighbor matching (caliper = 0.05) on propensity scores estimated from game genre, country (top 10), debut age, and debut year. SMD = Standardized Mean Difference (| SMD| < 0.10 indicates acceptable balance). *p*-values from Mann-Whitney U tests.

**FIGURE 6:**
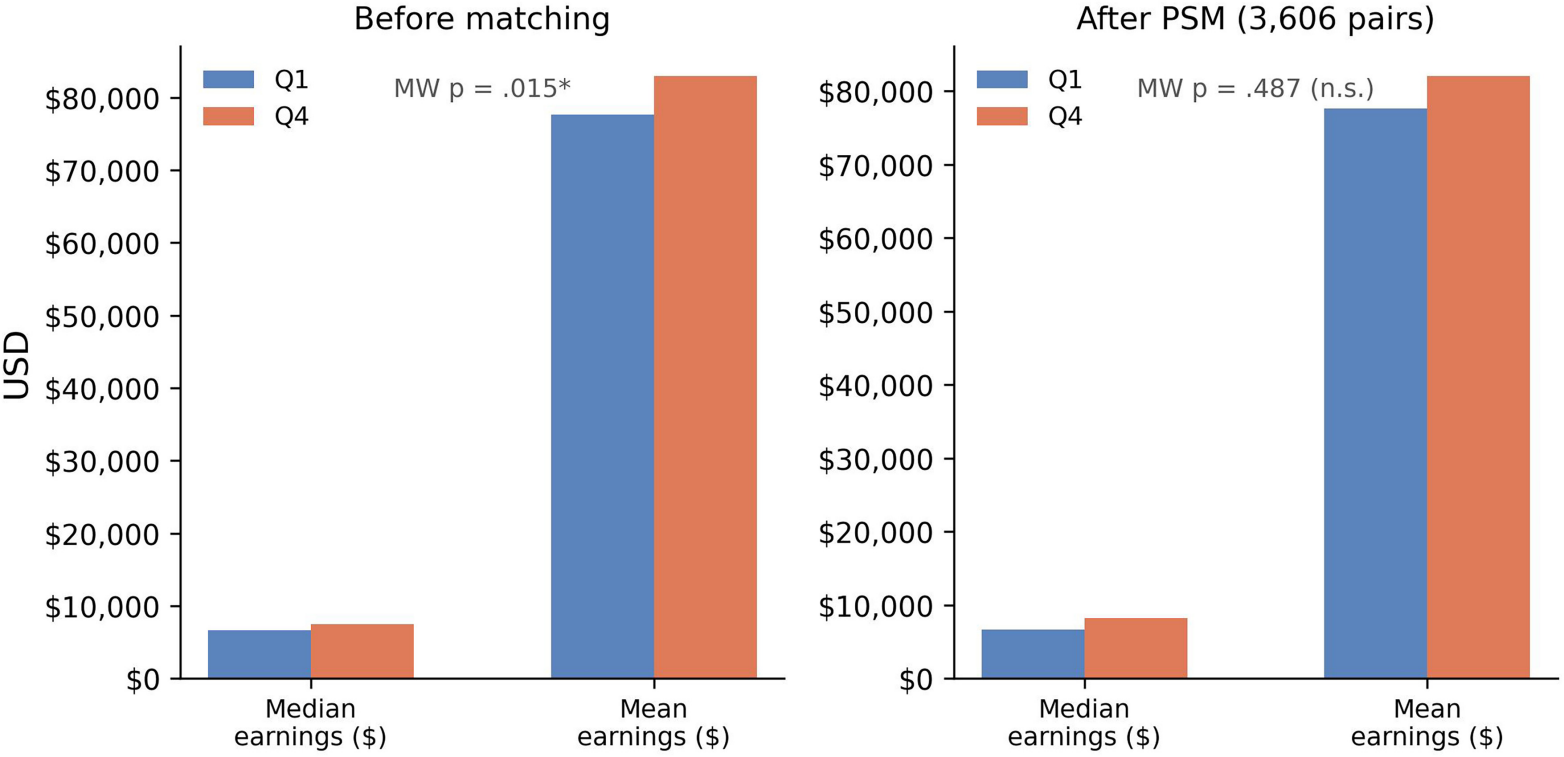
Propensity score matching comparison of Q1 versus Q4 career earnings. Left panel: before matching (full sample), the Q1–Q4 difference was marginally significant (Mann–Whitney *p* = 0.015). Right panel: after 1:1 nearest-neighbor matching on game genre, country, debut age, and debut year (3,606 matched pairs), the difference was non-significant (*p* = 0.487), indicating that the raw Q1 earnings disadvantage was attributable to compositional differences rather than a direct birth quarter effect. *p < 0.05.

In the matched sample, all outcome differences were non-significant (Figure 6). Median earnings were $6,668 (Q1) versus $8,217 (Q4), with a Mann–Whitney *p* = 0.487. Median career duration was 2.80 years (Q1) versus 3.10 years (Q4), *p* = 0.820.

As additional robustness checks, first-year earnings (the total prize money earned within 12 months of debut) were compared across birth quarters and did not differ at conventional significance levels (Kruskal–Wallis *H* = 5.87, *p* = 0.118). The second-year-to-first-year earnings growth ratio was also equivalent across quarters (*H* = 3.06, *p* = 0.382), ruling out differential early-career growth trajectories.

#### 3.3.4 Productivity within long-career survivors

A direct test of the underdog hypothesis (Gibbs et al., 2012; McCarthy and Collins, 2014) was conducted by restricting the analysis to players with career duration of 5 years or longer (*N* = 4,180; 27.7% of the main sample). Within this subset of career survivors, the birth quarter distribution was Q1 = 22.6%, Q2 = 25.2%, Q3 = 26.5%, Q4 = 25.8% [χ^2^(3) against uniform = 14.36, *p* = 0.002, Q1 vs. Q4 OR = 0.877], continuing the inverse-direction composition pattern observed in earlier subgroup analyses.

Four productivity metrics were then compared across birth quarters within this subset: total tournament prize earnings (Q1 median = $58,374, Q4 median = $60,560), earnings per active career year (Q1 = $6,523, Q4 = $7,334), tournaments per active year (Q1 = 4.6, Q4 = 4.4), and total tournament entries (Q1 = 35, Q4 = 31). None of the four metrics differed by birth quarter (Kruskal-Wallis *p* = 0.39–0.78 across metrics; Mann-Whitney Q1 vs. Q4 *p* = 0.18–0.79). Cohen’s d effect sizes for Q4 versus Q1 ranged from -0.017 to +0.027 across the four metrics, all negligible by conventional benchmarks (Cohen, 1988).

### 3.4 RQ4: does birth quarter work through debut age?

#### 3.4.1 Birth quarter differences in debut age

As noted in section 3.1.3, a systematic relationship between birth quarter and debut age was observed. Q1 players debuted at a mean age of 20.48 years, compared with 20.31 (Q2), 20.19 (Q3), and 20.17 (Q4). The one-way ANOVA was significant [*F*(3, 15,027) = 4.60, *p* = 0.003], as was the Q1 versus Q4 contrast (*t* = 3.30, *p* = 0.001; mean difference = 0.31 years). Logistic regression confirmed a monotonic trend: each year increase in debut age raised the probability of Q1 membership by 1.5% [OR = 1.015, 95% CI (1.006, 1.025), *p* = 0.001), and correspondingly lowered Q4 probability (OR = 0.991, *p* = 0.045).

#### 3.4.2 Mediation analysis

The Baron and Kenny mediation analysis is summarized in Table 7. The independent variable was BQ_late (1 = born July–December, 0 = born January–June), and all models controlled for game genre and team play.

**TABLE 7:** Mediation analysis: birth quarter → debut age → log earnings (Baron and Kenny framework with Sobel test).

Path/effect	β	SE	*P*
Step 1: Total effect (c)			
BQ_late → Log earnings	+0.066	0.041	0.102
Step 2: IV → Mediator (a)			
BQ_late → Debut age	–0.162	0.063	0.010**
Step 3: Direct + Mediator (c’ + b)			
BQ_late → Log earnings (c’)	+0.034	0.039	0.376
Debut age → Log earnings (b)	–0.198	0.005	< 0.001***
Indirect effect			
a × b	+0.032		*p* = 0.010*
% mediated	48.5%		

BQ_late = 1 if born July–December (Q3 or Q4), 0 otherwise. All models control for game genre (dummies) and team play. The indirect effect (a × b) represents the portion of the total association transmitted through the debut age pathway. BQ_late = 1 if Q3 or Q4 (born July–December), 0 otherwise. All models control for game genre (dummies) and team play. Indirect effect tested via Sobel test (Sobel *z* = 2.57). Genre and team play coefficients omitted for brevity.

*p < 0.05,

**p < 0.01,

***p < 0.001.

*Step 1 (total effect, path c):* The total effect of BQ_late on log earnings was positive but non-significant (β = +0.066, SE = 0.041, *p* = 0.102), indicating a small positive but non-significant association between late birth and earnings.

*Step 2 (path a):* BQ_late significantly predicted debut age (β = –0.162, SE = 0.063, *p* = 0.010), confirming that late-born players debuted approximately 0.16 years earlier than early-born players, controlling for genre and team play.

*Step 3 (paths b and c’)*: When debut age was added to the model, it was a strong negative predictor of log earnings (β = –0.198, SE = 0.005, *p* < 0.001), indicating that each additional year of debut age was associated with approximately 18% lower cumulative earnings. The direct effect of BQ_late was attenuated to β = +0.034 (*p* = 0.376), representing a 48.5% reduction from the total effect.

The indirect effect (a × b = +0.032) was statistically significant by the Sobel test (z = 2.57, *p* = 0.010), indicating that nearly half of the modest total association between late birth and higher earnings was transmitted through the debut age pathway.

#### 3.4.3 Interaction between birth quarter and debut age group

The two-way interaction between birth quarter and debut age group (early: < 18 years; mid: 18–22; late: > 22) in predicting log earnings produced two significant interaction coefficients: Q3 × mid-debut (β = –0.374, *p* = 0.004) and Q3 × late-debut (β = –0.386, *p* = 0.012). The joint *F*-test for all interaction terms was marginally significant (*F* = 1.96, *p* = 0.068). Interpretation of these interaction patterns is deferred to the Discussion (section 4.3).

#### 3.4.4 Team versus individual divergence

The debut age gradient operated differently depending on competitive structure. Among team-based players, those who debuted early ( < 18 years) showed a pronounced inverse RAE: Q1 = 22.7%, Q4 = 26.4% [χ^2^(3) = 15.88, *p* = 0.001]. This inverse pattern persisted, though attenuated, for mid-career debuts (18–22: Q1 = 23.5%, Q4 = 25.2%, *p* = 0.011) and vanished for late debuts ( > 22: *p* = 0.798). Among individual players, the pattern was reversed: early-debut players showed no significant BQ variation (*p* = 0.674), but late-debut players exhibited a significant traditional RAE (Q1 = 28.3%, Q4 = 23.6%, *p* = 0.013).

## 4 Discussion

### 4.1 Summary of findings

This study examined the relative age effect across 15,065 professional esports players from 177 countries, extending the existing two-study evidence base from the entry question to career trajectories, cross-national causal mechanisms, and mediation pathways. After adjusting for population birth seasonality, no aggregate RAE was detected [χ^2^(3) = 1.38, *p* = 0.711], though a striking cohort reversal emerged: players born after 2006 showed a strong traditional RAE (OR = 1.92), while those born in 1991–2000 showed a significant inverse pattern (OR = 0.81–0.85). The cross-national quasi-experimental analyses using 20 countries with different academic cutoffs revealed no causal effect of cutoff position on career outcomes (interaction F *p* = 0.886; OLS β = +0.004, *p* = 0.959). Conditional on reaching professional status, birth quarter had no direct effect on career longevity (log-rank *p* = 0.593; Cox HR ≈ 1.00) or earnings (PSM *p* = 0.487). Birth quarter did, however, operate indirectly through debut age: late-born players debuted 0.16 years earlier, and this earlier entry partially explained their marginally higher cumulative earnings (48.5% mediated; Sobel *z* = 2.57, *p* = 0.010).

### 4.2 Decomposing the RAE: what esports reveals about traditional sport mechanisms

The central theoretical contribution of this study lies not in documenting the absence of RAE in esports, a conclusion already suggested by Laxdal and Erikstad (2025), but in leveraging the structural uniqueness of esports to evaluate which observable birth-distribution patterns are consistent with each of the three traditional RAE mechanisms. A full mechanistic decomposition would require anthropometric and biological-maturation data not available in the present dataset; the analyses below therefore test which mechanisms remain plausible given the birth-quarter evidence, rather than directly measuring biological or psychological processes. The three mechanisms identified in the Introduction can now be evaluated in light of the present findings.

#### 4.2.1 Mechanism 1: physical maturation advantage

In traditional sports, relatively older children within an age cohort tend to be larger and more physically developed, giving them an advantage in selection contexts that reward size, strength, and speed (Cobley et al., 2009). Esports eliminates this mechanism by design: competitive success depends on cognitive-motor skills such as reaction time, decision-making speed, and strategic coordination, none of which are systematically linked to the few months of physical maturation that separate Q1 from Q4 players within the same birth year. The absence of any direct birth quarter effect on career outcomes in the present study, whether measured through survival analysis (section 3.3.1), earnings regression (section 3.3.2), or propensity score matching (section 3.3.3), is consistent with the hypothesis that physical maturation is the primary engine of RAE in traditional sports, and that removing it eliminates the direct effect.

#### 4.2.2 Mechanism 2: age-based selection structures

The most telling evidence against this mechanism comes from the cross-national quasi-experiment (section 3.2). If academic or sport cutoffs causally shape esports careers, as they do in traditional sports, where shifting the cutoff date shifts the birth-month advantage (Musch and Hay, 1999; Helsen et al., 2005), then the same birth month should produce different career outcomes depending on the player’s national cutoff system. Data are inconsistent with this prediction across multiple specifications. The OLS model with country fixed effects showed a near-zero coefficient for relative age, and the interaction between cutoff group and birth quarter was entirely non-significant (section 3.2). The Korean illustration, in which the same 1,216 players yielded an inverse RAE under a March cutoff and a traditional RAE under a January cutoff, demonstrated that the observed birth quarter distribution tracks baseline birth seasonality rather than any cutoff-driven selection mechanism. This null finding is consistent with the structural reality of esports: without age-grouped developmental pathways, there is no institutional mechanism through which a cutoff date could systematically advantage one birth month over another. When the same population yields opposite patterns under different cutoffs, the cutoff is a convention, not a mechanism.

Taken together, the nullification of Mechanisms 1 and 2 in esports provides indirect but compelling evidence for their centrality in traditional sport. The RAE literature has long argued that physical maturation and age-based selection jointly produce birth quarter disparities (Wattie et al., 2015), but because these mechanisms co-occur in conventional sport, their relative contributions have been difficult to quantify. Esports offers a setting where both are naturally “switched off,” and the result is consistent across analyses: once these two mechanisms are removed, no direct effect of birth quarter on career outcomes is detectable across the present analyses, within the precision afforded by the analytical sample.

#### 4.2.3 Mechanism 3: cumulative advantage and self-reinforcing dynamics

The third mechanism, the idea that early selection advantages compound over time through differential resource allocation, coaching quality, and motivational feedback (Hancock et al., 2013), operates differently in esports than in traditional sports, but it is not entirely absent. The mediation analysis (section 3.4) revealed that late-born players debut approximately 0.16 years earlier and that this earlier debut partially mediates a modest earnings advantage. This suggests a form of cumulative advantage that runs in the opposite direction from traditional sports: rather than early-born players accumulating advantages through preferential selection, late-born players in esports accumulate a slight edge through earlier self-initiated entry into competition. The effect is small, far smaller than the RAE observed in traditional sports, but its existence indicates that birth timing is not entirely irrelevant even in an open-access competitive system.

### 4.3 Self-selection versus structural selection: the open pathway

The debut age mediation finding illuminates a fundamental difference in how talent enters the competitive pipeline in esports versus traditional sports. In traditional sports, the pathway to elite competition is gate-kept: coaches, scouts, and academy administrators make selection decisions at each developmental stage, and these decisions are systematically biased toward relatively older, more physically mature athletes (Cobley et al., 2009; Hancock et al., 2013). This is a process of structural selection: the institution determines who advances.

In esports, the dominant pathway is the online ranked ladder, where players are matched by skill rating and progress through demonstrable performance rather than through selection by external evaluators. This is a process of self-selection: the individual determines when to enter and how far to advance. The finding that late-born players debut slightly earlier is consistent with a self-selection interpretation: in the absence of age-based gatekeeping, players enter professional competition when they judge themselves ready, and birth quarter may subtly influence this self-assessment through developmental timing. The relative age effect, on this view, is less a property of athletes than of the systems that select them.

The team-versus-individual divergence (section 3.4.4) provides further support for this interpretation. In team esports, where selection does involve organizational decision-making (tryouts, scouting reports, roster decisions), the entry process more closely resembles traditional sports, and the result is an inverse RAE among early-debut players. One plausible explanation is that team selectors in esports, unlike their counterparts in physical sports, evaluate candidates primarily through performance metrics derived from online play. In this context, relative maturity may offer no visible advantage, and relatively younger players who have developed advanced skills may stand out precisely because they achieved competitive performance at a younger absolute age. In individual esports, where entry is entirely self-directed, late-debut players showed a traditional RAE pattern, suggesting that early-born individuals who enter esports later in life may include those who initially pursued other activities, potentially including traditional sports, where their relative age conferred an early advantage, before transitioning to competitive gaming.

This analysis complicates the conclusion drawn by Laxdal and Erikstad (2025) that esports is simply “where age doesn’t matter.” A more precise formulation would be that esports is where age-based institutional selection does not operate, but birth timing still shapes career trajectories through the timing of self-initiated entry. The distinction matters theoretically: it suggests that the RAE in traditional sports is not a property of competitive domains *per se*, but a property of age-grouped selection systems. Remove the system, and the direct effect on birth-quarter distributions ceases to be detectable; what remains is the residual influence of developmental timing on individual decision-making.

An important qualification to this self-selection account is that esports is not selection-free; selection has been internalized into the within-game matchmaking rating (MMR) and skill-ranking systems that govern competitive ladders. MMR-based matchmaking continuously sorts players by demonstrated performance (Kang and Kim, 2026), and only those who climb sufficiently high in the rating distribution become visible enough to enter the Esports Earnings prize-record system. The analytical sample, then, is not the open population of competitive gamers but the survivor population of MMR-filtered competitors. This has two implications for the present results. First, any RAE operating at the MMR-filtering stage, for example, if early-born players were preferentially advanced through ranked tiers, would already have shaped sample composition before any of the analyses reported here. Second, the absence of a detectable birth-quarter effect within this filtered sample is consistent with two distinct interpretations: that no RAE operates at the MMR stage, or that MMR filtering produces enough underdog-style survivors to obscure any residual effect. Disentangling these would require pre-professional ladder data with linked birthdate information, which is not currently accessible at scale.

A related mechanism deserves consideration: cumulative advantage may operate not only within esports but also across competitive domains, through carry-over from prior athletic histories. The inverse RAE observed among adult cohorts could reflect, in part, the differential dropout pattern documented in traditional sports (Cobley et al., 2009; Hancock et al., 2013). Players born late in the year who were systematically underselected in traditional youth sport may have experienced early negative selection feedback that eroded motivation and self-efficacy in those settings, and may have subsequently redirected to open-access digital competition where accumulated skill and intrinsic motivation rather than relative maturity determine outcomes. In this reading, esports inherits a population that has been pre-filtered by the structural disadvantages of traditional sport, with the late-born overrepresented because they were pushed out elsewhere and motivated to seek competitive identity in a domain where the institutional barriers that disadvantaged them do not apply. This carry-over hypothesis is consistent with the cohort patterns observed in the present data and provides a Matthew-effect mechanism operating in reverse: institutional disadvantage in one domain generates compositional advantage in another.

An alternative interpretation, the underdog hypothesis, proposes that late-born athletes who persist past age-graded selection accumulate distinctive psychological resources, including resilience, intrinsic motivation, and adaptive problem-solving, that translate into superior long-term performance (Gibbs et al., 2012; McCarthy and Collins, 2014). In skill-based competitive environments such as esports, where outcomes depend on cognitive-motor performance rather than physical maturation, these accumulated capacities may continue to confer advantage. Underdog dynamics may also account for the team-individual divergence reported in the present data: in team selection contexts where decision-makers actively recruit, underdog signals may be amplified into recognition of demonstrated competence; in individual ladders where entry timing reflects self-assessment, the effect is muted. The underdog interpretation complements rather than displaces the cross-domain carry-over framing: both predict an inverse RAE concentrated among adults who entered esports before formalized youth pathways emerged.

The two framings make divergent predictions about within-subset productivity that can be tested directly in the present data. The carry-over hypothesis predicts compositional differences only: more late-born players reach the long-career subset, but they perform equivalently to their early-born peers once they arrive. The underdog hypothesis adds a quality prediction: within the survivor subset, late-born players should outperform early-born players because of accumulated psychological resources. Empirical examination of the 5+ year career subset reported in Section 3.3.4 supports the compositional prediction (Q1 vs. Q4 OR = 0.877, *p* = 0.002 against uniform) but rejects the quality prediction: all four productivity metrics show no birth quarter differences, with Cohen’s d values between –0.02 and +0.03. The data therefore favor the cross-domain carry-over framing over the within-individual underdog mechanism, though both processes may contribute jointly to the observed cohort patterns.

### 4.4 The paradox of formalization

The cohort reversal documented in section 3.1.2 carries implications that extend beyond the present dataset. Among players born after 2006 (the youngest cohort in sample, with a mean debut age of 15.7 years), the birth quarter distribution showed a pronounced traditional RAE (OR = 1.92, *p* < 0.001). This pattern is the mirror image of what was observed in older cohorts and stands in stark contrast to the aggregate null.

Although the small sample size (*N* = 449) warrants caution, the effect size is large by RAE standards (*V* = 0.112, compared with typical values of 0.02–0.05 in the broader esports sample and 0.05–0.15 in traditional sport meta-analyses; Cobley et al., 2009). The timing of this cohort’s emergence coincides with the rapid expansion of formalized youth esports structures over the past decade: organized youth leagues, esports-focused academies, and age-restricted competitive divisions have proliferated in markets such as South Korea, China, and North America (Kang, 2025; Kang and Kim, 2025). These structures increasingly resemble the age-grouped developmental pathways of traditional sports, and may be importing the same selection biases.

This finding suggests what might be called a paradox of formalization: the very process of professionalizing and institutionalizing esports, which aims to create structured developmental pathways, protect young players, and raise competitive standards, may inadvertently introduce the age-based selection biases that esports has historically avoided. If age-restricted youth leagues and academy tryouts become the standard entry pathway, replacing the open online ladder system, the conditions that currently suppress RAE in esports will erode. The result could be a gradual convergence between esports and traditional sports in the magnitude and direction of relative age effects. What esports avoided by remaining open, it now risks rediscovering by becoming structured.

This interpretation aligns with the broader trajectory of esports governance. Recent developments including the inclusion of esports in the Olympic Esports Games and the establishment of national esports federations have accelerated institutional formalization (Kang, 2025). Webdale et al. (2020), in their review of RAE mitigation strategies for traditional sports, proposed solutions such as rotating cutoff dates, expanding age bandwidths, and bio-banding by developmental status. The findings suggest that these solutions should be proactively considered for esports youth programs before traditional RAE patterns become entrenched, a preventive rather than corrective approach.

### 4.5 Methodological contributions

Beyond its theoretical contributions, this study offers several methodological advances for RAE research in esports and adjacent domains. First, the systematic documentation of the January 1st placeholder problem in the Esports Earnings database, and the three-tiered preprocessing protocol developed to address it, provides a reusable quality-assurance procedure for future studies relying on this data source. The finding that 2.0% of recorded birthdates were default placeholders, concentrated among players from countries with less developed information infrastructure, underscores the importance of data auditing prior to birth distribution analyses.

Second, the study demonstrates the critical importance of controlling for population birth seasonality when interpreting birth quarter distributions. The entire apparent inverse RAE in the aggregate sample [χ^2^(3) = 13.18, *p* = 0.004 against a uniform distribution] was eliminated once expected frequencies were adjusted for natural monthly birth variation [χ^2^(3) = 1.38, *p* = 0.711]. This discrepancy has direct implications for the two existing esports RAE studies, neither of which applied population-based corrections. More broadly, given that birth seasonality varies by hemisphere, country, and historical period (Buckles and Hungerman, 2013; Currie and Schwandt, 2013), future RAE studies, whether in esports or traditional sports, should report results against both uniform and population-adjusted baselines.

Third, the cross-national quasi-experimental design exploiting 20 countries with different academic cutoffs represents, to my knowledge, the first application of this approach to esports data. While similar designs have been employed in traditional sport (Musch and Hay, 1999; Helsen et al., 2005, 2012), the global reach and diverse country composition of professional esports make it particularly well-suited to this form of analysis.

The Korean data warrant separate methodological discussion within this contribution. The same 1,216 players produced an inverse adjusted RAE under a March cutoff (the system in effect for their academic cohort, as all had birth years before 2009) and a traditional adjusted RAE under a January cutoff. This contrast is methodological rather than substantive: the underlying data are unchanged across analyses, yet the choice of cutoff alignment determines the sign of the observed effect. When cutoff selection is arbitrary, or when multiple cutoffs operate in parallel (academic-year and sport-federation systems, for example), birth quarter analyses are vulnerable to specification-driven conclusions. Any cross-national RAE study that pools countries with different cutoff systems, or that selects a single cutoff *post-hoc*, should report sensitivity analyses across alternative cutoff specifications. The present analyses include this robustness step, and the consistent null across specifications strengthens confidence in the substantive conclusion.

Fourth, the combination of propensity score matching with mediation analysis provides a multi-method triangulation strategy for distinguishing direct from indirect birth quarter effects, an approach that could be productively extended to RAE research in traditional sport, where post-entry career effects remain understudied.

### 4.6 Limitations

Several limitations warrant consideration. First, and most consequentially, the date of birth was available for only 10.2% of all players in the Esports Earnings database. Players with known birthdates are demonstrably more prominent (higher earnings, more tournaments, longer careers) than the broader population, meaning that the findings are most directly generalizable to the upper tier of the professional esports ecosystem. It is possible that RAE patterns differ among lower-profile competitive players for whom biographical data are not publicly available. Future research could employ inverse probability weighting or Heckman selection models to assess the sensitivity of conclusions to this selection process.

Two specific sensitivity analyses are particularly worth pursuing in future work. An inverse probability weighting approach could model disclosure probability as a function of country, debut year, career-stage indicators, and game-title fixed effects, with the resulting stabilized weights applied to the primary analyses. Such weighting would address the most plausible selection mechanism, that disclosure tracks public visibility, while preserving the analytical structure of the present study. A complementary Heckman selection model could jointly estimate disclosure and outcome equations, treating country and game-genre fixed effects as identification restrictions in the selection equation. Both approaches require auxiliary data on undisclosed players (country, debut tournament, career-record summaries) that were not retrieved in the present extraction protocol; their implementation is therefore left to future work that prioritizes the selection question.

Second, the population birth seasonality adjustment relied on a two-country average (United States and South Korea) rather than country-specific monthly birth distributions for all 177 nations in the sample. While this approximation is reasonable given that these two countries account for a substantial share of the professional esports population and exhibit birth seasonality patterns broadly representative of the Northern Hemisphere, it may introduce imprecision for players from countries with markedly different birth timing patterns (e.g., Southern Hemisphere or tropical nations). Country-specific birth statistics from national statistical agencies would strengthen future analyses.

Third, the 2006+ cohort finding, though statistically significant and substantively compelling, rests on a relatively small sample (*N* = 449). As this cohort matures and more players accumulate competitive records, the robustness of the observed traditional RAE can be reassessed longitudinally. At present, the finding should be regarded as exploratory and hypothesis-generating rather than definitive.

Fourth, career outcomes were operationalized exclusively through tournament prize earnings and career duration. Important dimensions of esports performance, such as in-game statistics (win rates, kill-death ratios, rating points), streaming revenue, and team salary, were not captured in the Esports Earnings database. Integrating these additional outcome measures would provide a more complete picture of how birth quarter does or does not influence career success.

Fifth, the mediation analysis, while informative, rests on observational data and the Baron and Kenny (1986) framework, which assumes no unmeasured confounding of the mediator–outcome relationship. While the cross-national null (section 3.2) provides corroborating evidence that no omitted cutoff-related mechanism is at work, the possibility of residual confounding in the debut-age pathway cannot be definitively excluded. Experimental or instrumental-variable approaches would strengthen causal inference, though such designs are challenging in this context.

Sixth, the analyses rely entirely on date-of-birth information; anthropometric characteristics, biological age, and maturation status were not available in the Esports Earnings database. The mechanistic discussion in section 4.2 is therefore interpretive rather than directly empirical: the present data can show whether birth-quarter distributions are consistent with each mechanism’s predictions, but cannot directly test the underlying biological or psychological processes that the maturation, structural-amplification, and dropout mechanisms posit. A full mechanistic decomposition would require linked anthropometric and developmental data that no public esports dataset currently provides.

Seventh, the analytical sample is composed of players who have already passed through within-game matchmaking-rating (MMR) and skill-ranking filters, as discussed in section 4.3. The methodology applied here assesses birth-quarter distributions among MMR-filtered prize-winning competitors, not within the upstream amateur or recreational populations from which the professional pool is drawn. Any RAE operating at the MMR-filtering stage would already be encoded in the sample, and the present null results should be interpreted as referring to the post-MMR professional segment of the population rather than to esports participation as a whole.

Eighth, regarding generalizability (STROBE Item 21), the findings apply most directly to the upper tier of birthdate-disclosing professional esports competitors active across 177 countries between 1997 and 2025, predominantly concentrated in North America, East Asia, and Western Europe. The results should not be extrapolated to amateur esports populations, to recreational competitive gamers, or to the substantial fraction of professional players whose birthdates remain undisclosed. Generalization to esports titles or game genres not represented in the present sample (e.g., emerging mobile-only or location-based augmented-reality games) is similarly cautious. Within the analytical sample, the cross-national, multi-genre, multi-cohort coverage supports moderate-confidence generalization across major globally documented esports titles.

Finally, the retirement definition (two or more years since last tournament entry) is inherently arbitrary. Sensitivity analyses using 1- and 3-year thresholds produced substantively identical results for all birth quarter comparisons, but the absence of official retirement declarations in esports means that some classified “retirees” may in fact be on extended competitive breaks.

### 4.7 Future directions

Three directions for future research follow naturally from the present findings. First, longitudinal tracking of the 2006+ cohort as it matures will be essential to determine whether the traditional RAE observed among the youngest entrants persists, intensifies, or attenuates as these players accumulate competitive experience. If the paradox-of-formalization hypothesis is correct, the effect should strengthen in regions and game titles where structured youth programs are expanding most rapidly.

Second, the integration of game-internal performance data (reaction time metrics, actions-per-minute, skill ratings) would allow researchers to test whether birth quarter influences the cognitive-motor capacities that underpin esports performance, independent of career-level outcomes. Thompson et al. (2014) demonstrated that cognitive-motor decline begins around age 24 in StarCraft 2 players, but whether this age-linked decline interacts with relative age within birth cohorts remains unexplored.

Third, direct comparison between esports academy participants and open-ladder entrants within the same game ecosystem would provide the most targeted test of whether age-based selection structures are the mechanism responsible for the emerging RAE in the youngest cohort. If academy entrants show a significant RAE while same-aged ladder entrants do not, the case for the paradox of formalization would be substantially strengthened.

## 5 Conclusion

This study set out to determine whether the relative age effect, one of the most robust phenomena in developmental sport science, operates in professional esports, and if so, through what mechanisms. By analyzing 15,065 players across 177 countries and 609 game titles, and by moving beyond the entry-level analysis of prior work to examine career trajectories, cross-national causal pathways, and mediation mechanisms, a set of conclusions emerges that both confirm and substantially extend the existing evidence base.

At the aggregate level, there is no relative age effect in professional esports once population birth seasonality is accounted for. The apparent overrepresentation of later-born players reported in prior studies reflects the natural seasonal pattern of human births, not a selection mechanism. This finding validates and refines the conclusion of Laxdal and Erikstad (2025), while demonstrating that the methodological step of adjusting for birth seasonality is not merely a refinement but a correction that eliminates a spurious effect.

Beneath this aggregate null, however, lies meaningful heterogeneity. A cohort reversal (inverse RAE among adult professionals born in the 1990s, traditional RAE among the youngest entrants born after 2006) replicates the age-stratified pattern first observed in Swedish data by Jakobsson et al. (2021) and extends it to a global, multi-game sample. The cross-national quasi-experiment establishes that this variation is not caused by academic cutoff dates: the same birth month produces equivalent career outcomes regardless of its position within different national cutoff systems. The career trajectory analyses confirm that, once a player reaches professional status, birth quarter exerts no direct influence on how long or how lucratively they compete.

What birth quarter does influence, modestly but significantly, is the timing of entry. Late-born players debut approximately 2 months earlier, and this earlier debut partially mediates their marginally higher career earnings through a self-selection pathway that is structurally distinct from the institutional gatekeeping that drives RAE in traditional sports. The implication is that the RAE, as observed in traditional sports, is not a universal property of competitive achievement domains but rather a specific consequence of age-grouped selection systems. Remove those systems, and the direct effect on birth-quarter distributions ceases to be detectable within the precision afforded by the present analyses.

The emergence of a traditional RAE among the youngest cohort raises a cautionary flag. As esports increasingly adopts the institutional trappings of traditional sports (age-restricted youth leagues, formalized academies, structured tryout processes), it risks importing the very selection biases it has historically avoided. The paradox of formalization suggests that stakeholders in esports governance should proactively adopt RAE-aware policies, drawing on the mitigation strategies developed over four decades of traditional sport research, before these patterns become entrenched.

More broadly, this study demonstrates the value of esports as a theoretical testing ground for sport science. The absence of physical maturation advantages and age-based selection structures in esports creates a natural experiment that allows researchers to isolate mechanisms that are confounded in traditional sports. As esports continues to grow in scale, institutional complexity, and data availability, it offers an increasingly powerful lens through which to examine fundamental questions about talent development, selection bias, and the structural determinants of competitive careers. Where an effect does not appear, what produces it elsewhere is most clearly seen.

## Data Availability

The raw data supporting the conclusions of this article will be made available by the authors, without undue reservation.

## Supplementary material

Supplementary Material S1 Reproducibility package containing the complete analytical pipeline, the preprocessed analytical samples, per-phase analytical outputs, and a verification report documenting reproduction of all statistics reported in the manuscript.
